# Effect of Statins on Major Adverse Cardiovascular Events in Patients with Coronary Artery Spasm: A Meta-Analysis of the Asia Region

**DOI:** 10.1155/2023/8807278

**Published:** 2023-04-27

**Authors:** Tian-Jun Zhao, Duan Luo, Xi Jiang, Feng Tang, Hui Jiang

**Affiliations:** ^1^Department of Cardiology, The Affiliated Hospital of Southwest Jiaotong University, The Third People's Hospital of Chengdu, Cardiovascular Disease Research Institute of Chengdu, Chengdu 610031, China; ^2^Department of Gastroenterology, The Affiliated Hospital of Southwest Jiaotong University, The Third People's Hospital of Chengdu, Chengdu 610031, China

## Abstract

**Background:**

Whether statins can reduce major cardiovascular adverse events (MACE) in patients with coronary artery spasm (CAS) is controversial. And most of the relevant research to date has been conducted in Asia.

**Methods:**

We systematically searched electronic databases for studies on the effect of statins on MACE in patients with CAS in Asia and published up to September 2022. We included data on MACE in a statin therapy patient group and a no-statin therapy control group. We then evaluated the effect of statin therapy on MACE in patients with CAS in Asia by meta-analysis and trial sequential analysis (TSA). All statistical analyses were performed using Stata 16.0 software and TSA software.

**Results:**

A total of 10 studies (*n* = 9333 patients) were included in the final analysis. Meta-analysis showed that the use of statins had a significant effect on MACE in CAS patients (with RR, 0.70; 95% CI, 0.49-0.99), and the sensitivity analysis further confirmed this finding. Subgroup analysis suggested that the correlation between statin therapy and reduced MACE endpoint was stronger in Japanese patients and patients followed up for more than 4 years. But our TSA results indicated that the available samples were insufficient and further research is needed.

**Conclusions:**

Our meta-analysis suggests that statin therapy can reduce MACE in patients with CAS in Asia, and the correlation between the two was stronger in Japanese patients and patients followed up for more than 4 years.

## 1. Introduction

Coronary artery spasm (CAS) is known as focal or generalized vasospasm of coronary arteries and has a higher prevalence in Asian patients [1]. Although the exact mechanism of CAS has not been fully determined, some factors such as vascular inflammation, autonomic nerve dysfunction, vascular smooth muscle hyperresponsiveness, coronary artery microvascular dysfunction, and endothelial dysfunction can affect vasospasm [1]. It can occur in patients with or without atherosclerosis and influence epicardial coronary arteries or microvascular systems. CAS is an under-understood factor of acute coronary syndrome, stable angina pectoris, malignant arrhythmias, and sudden cardiac death [2].

Although drugs of CAS, including calcium channel blockers (CCBs) and nitrates, have been shown to be highly effective in suppressing coronary spasm, some patients are still at risk of major adverse cardiovascular events (MACE) [3]. Statins (3-hydroxy-3-methylglutaryl coenzyme A reductase inhibitors) can reduce atherosclerotic vascular events and are widely used in patients with coronary artery disease (CAD). There is increasing evidence that statins are related not only to lipid-lowering effects and plaque regression but also to other abilities such as improving endothelial dysfunction and reducing vascular inflammation [4, 5].

Due to the multiple effects of statins, it is possible that statins may suppress coronary spasm and reduce adverse cardiovascular events. However, the effects of statin therapy on reducing adverse cardiovascular events in CAS have been inconsistently demonstrated in previous studies [6–11]. Additionally, most of the research to date has been conducted in Asia. Therefore, in this study, we conducted a meta-analysis to evaluate the effect of statins on MACE in patients with CAS in Asia.

## 2. Materials and Methods

### 2.1. Search Strategy

The meta-analysis was performed in strict accordance with the PRISMA (Preferred Reporting Items for Systematic Reviews and Meta-Analyses) guidelines [12]. The study protocol was registered in the International Prospective Register of Systematic Reviews (PROSPERO—registration number: CRD42022355491). According to the PRISMA guidelines, we systematically searched online databases (PubMed, Embase, and the Cochrane Library) before September 2022. The search terms were (atorvastatin OR fluvastatin OR lovastatin OR pitavastatin OR pravastatin OR rosuvastatin OR simvastatin OR statin OR statins OR “Hydroxymethylglutaryl-CoA Reductase Inhibitors”) AND (vasospastic angina OR variant angina OR spastic angina OR prinzmetal OR coronary spasm OR coronary vasospasm OR Coronary artery spasm OR Vasospasm OR “Angina Pectoris, Variant”).

### 2.2. Inclusion Criteria

Identified studies were enrolled based on the following inclusion criteria: (1) the study reported VSA patients in both the statin group and the no-statin group. (2) The clinical outcomes included major adverse cardiovascular events (e.g., cardiac death, acute myocardial infarction, and unstable angina). (3) The study reported a sample size and the number of endpoint events for a statin group and a no-statin group. (4) Both cohort studies and randomized controlled trials (RCTs) were eligible.

### 2.3. Exclusion Criteria

In the process of literature screening, studies including the following items are excluded: (1) experimental animal studies; (2) case-control studies, case reports, conference abstracts, review papers, editorials, commentaries, and small case series (*n* < 50); and (3) non-Asia regional studies or non-English literature.

### 2.4. Assessment of Study Quality

The quality of each study was evaluated by the Newcastle-Ottawa scale, which is a commonly used quality rating standard in cohort studies. The scoring system consisted of three parts (population selection, intergroup comparability, and exposure factors). The results ranged from 0 to 9, and the higher the score, the better the quality of the methodology.

### 2.5. Data Extraction

Each of the eligible articles was extracted by two researchers solely for the following characteristic: first author's name, year of publication, country, study population size, age, sex, hypertension, diabetes mellitus, dyslipidemia, smoking, and medications used for treatment (aspirin, calcium-channel blocker, nitrate, nicorandil, *β*-blockers, angiotensin-converting enzyme inhibitors, and angiotensin receptor blockers). We also extracted data regarding follow-up time of patients and MACE (including cardiac death, myocardial infarction, and unstable angina pectoris).

### 2.6. Statistical Analysis

For each study, we pooled the relative risk (RR) and their 95% confidence intervals (95% CI). The *I*^2^ test and Cochran *Q* statistics (chi-square test, *χ*^2^) were calculated to assess the heterogeneity. When *P* < 0.05 or *I*^2^ > 50%, it indicated that statistical heterogeneity was significant in the study. If there was a significant difference in heterogeneity, the random-effect model would be adopted to pool the RR value; or else, a fixed-effect model would be applied. We used the symmetry of a funnel plot to evaluate possible small sample effects, and Begg's test, as well as Egger's test, was used to evaluate publication bias in the included studies. Subgroup analysis was also performed based on country, study population size, and follow-up time. At the same time, sensitivity analysis was executed to examine the impact of individual studies on the total merged effects to assess the reliability of the conclusions. To reduce the risk of type I error and estimate the required information size (RIS) needed to achieve a preset power level, we conducted the trial sequential analysis (TSA) with a 5% risk of a type I error and a power of 80%. All statistical tests were two-sided, and *P* < 0.05 was considered as statistically significant. All statistical analyses were performed using Stata 16.0 software and TSA software.

## 3. Results

### 3.1. Description of the Studies

**Figure 1:**
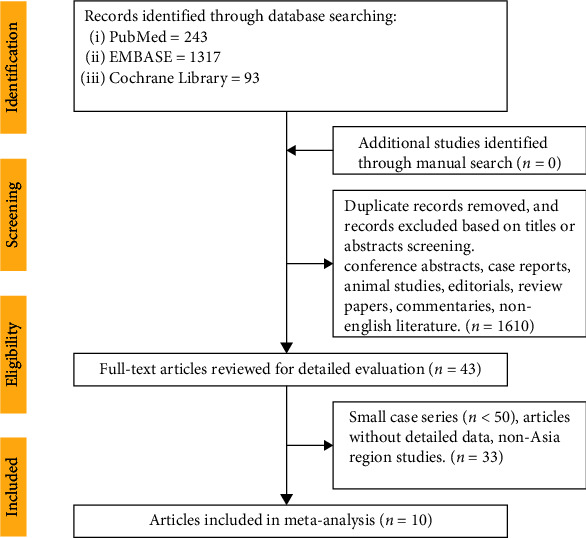
Flowchart of selected articles.

After screening 1653 titles and abstracts, 43 studies were reviewed for detailed evaluation in full text, of which 10 adhered to our inclusion criteria and were selected for this meta-analysis [6–11, 13–16]. The flowchart for the inclusion and exclusion process is shown in further detail in Figure 1. The meta-analysis included 4345 patients in the statin group and 4988 controls in the no-statin group, and the number of patients in each study ranged from 231 to 4099. These 10 studies were published between 2007 and 2022. Three of the studies were examined by the method of propensity matching. One of the studies was conducted in the intensive care unit, and one study was performed in patients with coronary spasm-induced acute myocardial infarction. The main features and clinical outcomes of the studies are shown in Tables 1 and 2.

### 3.2. Risk of Bias Assessment

Of the 10 studies, except for 1 case-control study, 9 were cohort studies, of which 2 were prospective and 7 retrospective studies. Considering that there is only one case-control study, all studies were scored identically using the Newcastle-Ottawa tool. All studies scored greater than five and were included in the meta-analysis. A summary of the risk of bias assessments is presented in Table 3.

### 3.3. Outcomes Comparing Statin Therapy with No-Statin Therapy

#### 3.3.1. Meta-Analysis

**Figure 2:**
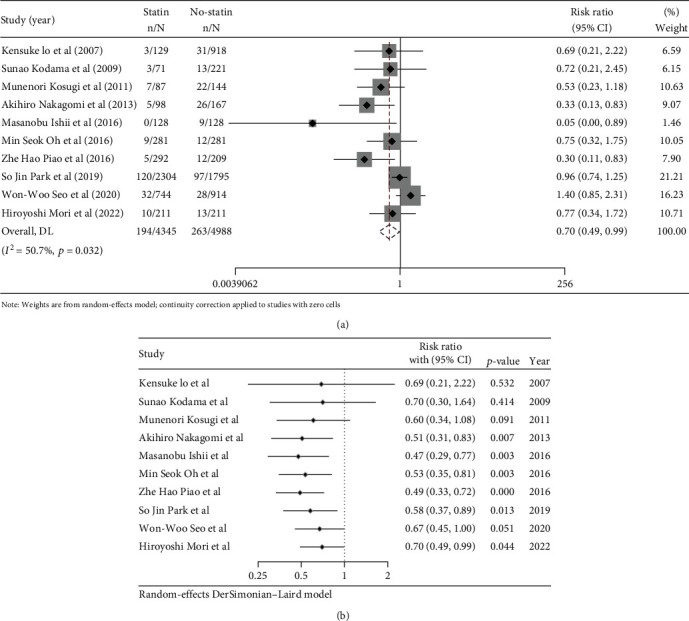
MACE in CAS patients between the statin therapy patient group and the no-statin therapy control group: (a) meta-analysis; (b) cumulative meta-analysis.

Meta-analysis showed that the use of statins had a significant effect on the incidence of cardiovascular events in CAS patients (with RR, 0.70; 95% CI, 0.49-0.99). Among the enrolled studies, significant heterogeneity was observed (*I*^2^ = 50.7%, *P* = 0.032). As a result, the random-effect model was used for the analysis of results in the present study. The forest plot results are shown in Figure 2(a). The cumulative meta-analysis can show the pattern of evidence over time and can identify the point when a treatment becomes clinically significant. As can be seen from Figure 2(b), statins may have a potential benefit in patients with CAS since 2013.

#### 3.3.2. Detection of Publication Bias and Sensitivity Analysis

**Figure 3:**
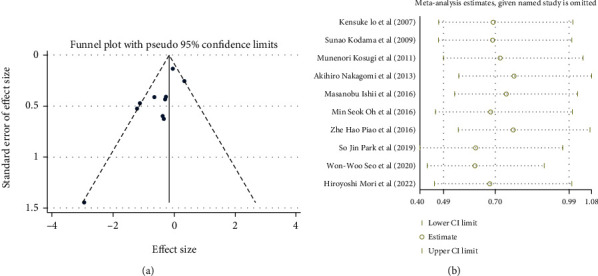
(a) Evaluation of publication bias by funnel plot. (b) Sensitivity analysis of the included studies.

The publication bias included in the included studies may have affected the results. Therefore, in the included studies, we used funnel chart, Begg's test, and Egger's test to assess potential publication bias. The funnel chart showed asymmetry, suggesting publication bias (Figure 3(a)). Egger's (*P* = 0.019) and Begg's (*P* = 0.107) tests were used to analyze the data, and the results showed that there may have obvious publication bias. We performed a sensitivity analysis by sequentially omitting individual studies and observed that our meta-analysis is statistically stable (Figure 3(b)).

#### 3.3.3. Subgroup Analyses

**Figure 4:**
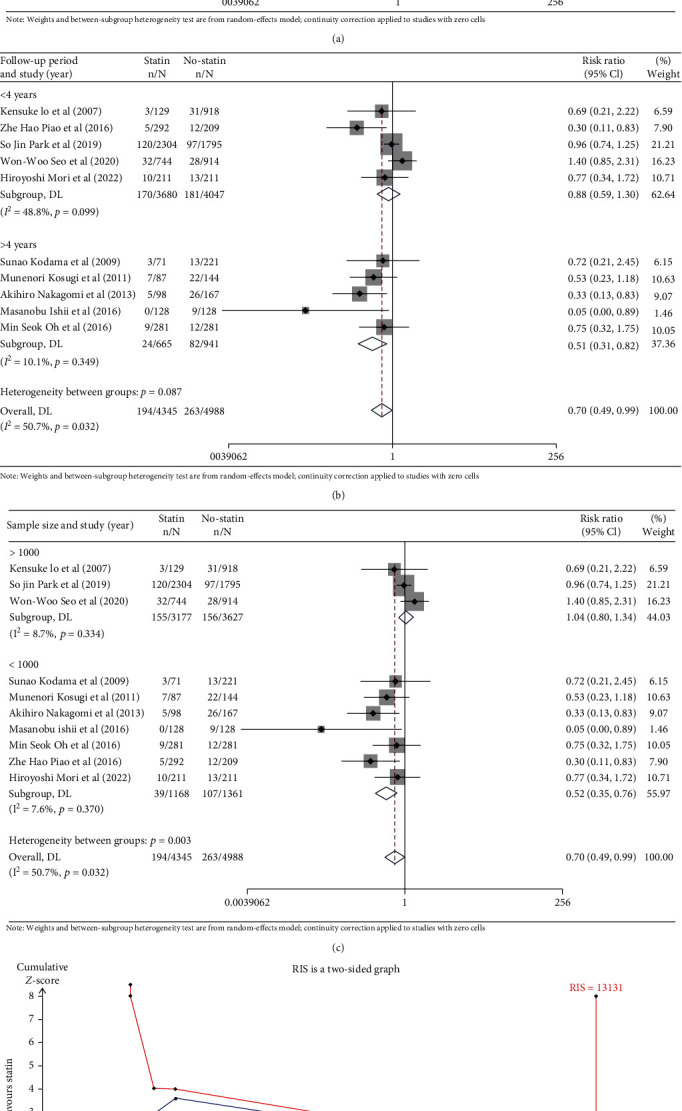
(a) Subgroup analysis by country. (b) Subgroup analysis by follow-up period. (c) Subgroup analysis by sample size. (d) Trial sequential analysis.

To reduce the influence of confounding factors on the results of this study, we conducted three subgroup analyses. We divided the included studies into the Japan group and the South Korea group according to the difference in country, divided the included studies into a <4-year group and a >4-year group according to the difference in follow-up period, and divided the included studies into a >1000 group and a <1000 group according to the difference in sample size. The subgroup analysis indicated that there was heterogeneity in the South Korea group (*n* = 4) (RR: 0.88; 95% CI: 0.56-1.38) (*I*^2^ = 60.2%, *P* = 0.057, Figure 4(a)), but the Japan group (*n* = 6) (RR: 0.54; 95% CI: 0.36-0.82) (*I*^2^ = 0.0%, *P* = 0.436, Figure 4(a)), the <4-year group (*n* = 5) (RR: 0.88; 95% CI: 0.59-1.30) (*I*^2^ = 48.8%, *P* = 0.099, Figure 4(b)), the >4-year group (*n* = 5) (RR: 0.51; 95% CI: 0.31-0.82) (*I*^2^ = 10.1%, *P* = 0.349, Figure 4(b)), the >1000 group (*n* = 3) (RR: 1.04; 95% CI: 0.80-1.34) (*I*^2^ = 8.7%, *P* = 0.334, Figure 4(c)), and the <1000 group (*n* = 7) (RR: 0.52; 95% CI: 0.35-0.76) (*I*^2^ = 7.6%, *P* = 0.370, Figure 4(c)) showed a significant decrease. Moreover, statins were significantly associated with a reduced risk of cardiovascular events in CAS patients in the Japanese group, the >4-year group, and the <1000 group.

#### 3.3.4. Trial Sequential Analysis

In the TSA, the diversity-adjusted information size of 13,131 patients was calculated using a two-side *α* = 5%, *β* = 20% (power 80%), an anticipated relative risk reduction of 30%, and an event proportion of 7.2% in the control arm. The result showed that the cumulative *Z*-curve crossed the conventional boundary for favoring statin therapy but did not surpass the trial sequential alpha spending monitoring boundary (Figure 4(d)).

## 4. Discussion

This meta-analysis included 10 studies that contained 4345 patients in the statin group and 4988 controls in the no-statin group. Our analysis showed that statin therapy can reduce adverse cardiovascular events in patients with CAS in Asia, and the sensitivity analysis further confirmed this finding. Additionally, a subgroup analysis suggested that statins were significantly associated with a reduced risk of MACE in CAS patients with an average follow-up of >4 years. Nevertheless, our TSA results indicated that the available samples were insufficient and further research is needed.

Currently, most of the studies on the effectiveness of statins for CAS have been conducted in Asia, particularly in Japan. Epidemiological data show that the prevalence of CAS varies between countries, and the frequency of CAS appears to be higher in the Japanese population than in western populations [17]. Furthermore, the frequency of multiple spasms (≥2 spastic coronary arteries) was significantly higher in the Japanese (24.3%) and Taiwanese (19.3%) populations (7.5%) than in the Caucasian population (7.5%) [18–20]. Studies of coronary artery pathophysiology have shown that Japanese patients have diffuse hyperreactive coronary vessels compared to white patients [17]. The mechanism responsible for this phenomenon remains to be elucidated, but both genetic and environmental factors may play a role. The influence of genetic factors has been paid attention to. Nitric oxide (NO) metabolism is closely related to vascular regulation mechanism, and eNOS gene is a main regulatory gene for NO metabolism in the vascular system. Tanus-Santos et al. demonstrated that eNOS alleles are unevenly distributed across ethnic groups [21]. Thus, ethnic diversity may partly explain the comparative results of genetic and clinical studies in predominantly white or Asian populations.

In recent years, research on the relationship between statin therapy and MACE in CAS patients has gradually increased. There were two prior meta-analyses that have studied the association between statin therapy and prognosis in CAS patients [22, 23]. These two studies indicated that no association was observed between statin use and reduced risk of MACE in patients with CAS. However, the meta-analysis by Liu et al. only included five studies, while Sayed et al. enrolled 9 studies including conference abstracts. Neither of these meta-analyses performed TSA; RIS cannot be evaluated. Through detailed search, our meta-analysis included 10 studies excluding conference abstracts, and RIS was calculated in our TSA. By including more studies, it is now possible to further examine subgroup analyses such as follow-up period, sample size, and country where the study was conducted.

Based on the sensitivity analysis results, we observed that none of the studies affected the overall effect, indicating that our meta-analysis is statistically stable. But results analyzed in the paper by Seo et al. are certainly a cause for concern [10]. The study included 1658 patients who were diagnosed with CAS based on coronary provocation test and excluded patients who had a history of percutaneous coronary intervention and stroke/transient ischemic attack and ≥50% obstructive CAD. The primary outcome was a composite of cardiac death, acute coronary syndrome, and new-onset life-threatening arrhythmia during a 3-year follow-up period. Their results show that statin therapy was not (HR, 1.35; CI, 0.78–2.33, *P* = 0.281) an independent predictor of primary outcomes in multivariate Cox regression analysis, nor were CCB (HR, 0.70; CI, 0.31–1.55, *P* = 0.378) and nitrates (HR, 0.69; CI, 0.38–1.27, *P* = 0.235). Seo et al. believe that the main reason for the lack of benefits of statins in the treatment of CAS may be that most of the selected patients have received CCBs or nitrates before discharge in this study.

Our analysis showed that the beneficial effect of statin therapy in patients with CAS in Asia despite TSA results indicated that the available samples were insufficient. The mechanisms by which statins could decrease MACE in patients with CAS are worthy of further study. It is generally known that statins can reduce cardiovascular events in patients with coronary heart disease by lowering LDL cholesterol levels and protecting against coronary artery plaque formation. In our meta-analysis, some studies included patients with mild to moderate organic coronary artery stenosis; except for those without coronary artery stenosis, these patients may have reaped greater benefits of antiatherosclerotic progression from statins [6–8, 10, 11]. Additionally, experimental and clinical evidences suggest that statins improve endothelial dysfunction through antioxidation, anti-inflammation, and increased production of endothelial nitric oxide (NO) [24–27]. Previous studies have shown that vascular smooth muscle hyperreactivity plays a key role in the pathogenesis of CAS, and the main reason seems to be the increased activity of Rho kinase [28, 29]. Statins have been shown to block the activation of RhoA, thereby improving vascular endothelial function, enhancing NO activity, and inhibiting inflammation and Ca^2+^ sensitivity of coronary smooth muscle [30, 31]. Finally, substantial overlap exists between patients with coronary microvascular dysfunction (CMVD) and CAS (32.6% in Suda et al. study and 20.5% in the CORMICA trial) [32, 33]. Of the 10 studies included in the present meta-analysis, the degree of overlap is unknown. A study consisted of 925 patients showed that statin treatment was associated with decreased MACE in CMVD patients over a long-term period (more than 10 years) [34].

The results of our subgroup analysis suggest that the correlation between statin therapy and reduced MACE endpoint was stronger in Japanese patients. The ethnic heterogeneity of coronary artery vasomotor reactivity does exist, but studies have mainly focused on differences between Japanese and Caucasian patients [17]. Caucasian variant angina patients not only have a higher incidence of atherosclerotic disease than Japanese but also have more extensive disease [35]. And Caucasian variant angina patients have a worse overall survival than their Japanese counterparts [17]. Genetic factors contributing to racial heterogeneity have been implicated, particularly with a focus on amino acid substitution of endothelial nitric oxide synthase gene (resulting in insufficient production of nitric oxide). The missense mutations are more frequent in Japanese patients with vasospastic angina than in the control group [36]. However, the low frequency of the gene in CAS suggests that other factors play a role. Future studies of Japanese patients living in Caucasian societies may further help us understand the environmental factors that contribute to this phenomenon. Additionally, statins were significantly associated with a reduced risk of MACE in CAS patients with an average follow-up of >4 years. We can speculate that the longer the statin is used, the greater the benefit for CAS patients. Some CAS patients have mild to moderate organic coronary artery stenosis and therefore benefit from the concept of “the lower the better” for LDL cholesterol. There is no doubt that prolonged statin therapy can significantly lower LDL cholesterol and thus reduce cardiovascular events. Many studies also have confirmed the importance of long-term cholesterol-lowering therapy for almost all patients with or without previous coronary heart disease events and support a longer-term view in determining the net benefits of treatment [37–39]. Due to the limitations of this meta-analysis, it is not possible to determine how long statin use reduces cardiovascular time in patients with CAS. This question is an interesting topic that deserves further study.

In the present analysis, our TSA results indicated that the available samples were insufficient. As visualized in the forest plot of the cumulative meta-analysis (Figure 2(b)) and the plot of TSA, we observed that previous trend has changed since the study by Park et al. [9]. In particular, the relatively large sample size of Park et al. study and Seo et al. study may lead to uncertainty in the link between statin therapy and MACE endpoints in CAS patients. In short, despite our analysis showing that statin therapy can reduce MACE in patients with CAS in Asia, this association appeared to be unstable. Therefore, more trials are required in this field, especially randomized controlled trials.

We should consider some potential limitations of this meta-analysis. First, there is the absence of randomized controlled trials in these studies included in this meta-analysis, and only 3 studies provided propensity score-matched analysis data. Second, the sample size of this study is too small to further confirm our findings. Finally, there are limited studies in some subgroup analysis, and more studies are needed to support these results. Although this study has a few limitations, it still provides some implications for statins in the treatment of CAS patients in Asia, which may inspire future research explorations and aid in the formulation of new hypotheses.

## 5. Conclusion

In general, our meta-analysis suggests that statin therapy can reduce MACE in patients with CAS in Asia; the correlation between the two was stronger in Japanese patients and patients followed up for more than 4 years. However, due to the insufficiency of available samples in this study, more studies are needed to confirm the future use of statins in CAS patients.

## Figures and Tables

**Table 1:** Main characteristics of included studies (statin group vs. no-statin group, number (%)).

Study	Hypertension	Diabetes mellitus	Dyslipidemia	Smoking	Aspirin	CCB	Nitrate	Nicorandil	*β*-Blockers	ACEIs/ARBs
Kensuke Io et al.	NR									
Sunao Kodama et al.	NR									
Munenori Kosugi et al.	NR									
Akihiro Nakagomi et al.	NR									
Masanobu Ishii et al.	60 (46.9) vs. 53 (41.4)	26 (20.3) vs. 21 (16.7)	121 (94.5) vs. 50 (39.1)	50 (39.1) vs. 51 (39.8)	37 (28.9) vs. 35 (27.3)	122 (95.3) vs. 124 (96.9)	14 (10.9) vs. 19 (14.8)	9 (7.0) vs. 9 (7.0)	8 (6.3) vs. 6 (4.7)	34 (26.6) vs. 29 (22.7)
Min Seok Oh et al.	110 (39.1) vs. 111 (39.5)	71 (25.4) vs. 73 (26.0)	NR	89 (31.7) vs. 82 (29.2)	180 (64.1) vs. 179 (63.7)	274 (97.5) vs. 274 (97.5)	108 (38.4) vs. 105 (37.4)	102 (36.3) vs. 97 (34.5)	NR	50 (17.8) vs. 49 (17.4)
Zhe Hao Piao et al.	122 (41.9) vs. 74 (35.6)	34 (11.7) vs. 21 (10.1)	29 (10.0) vs. 11 (5.3)	156 (53.8) vs. 115 (55.3)	229 (80.4) vs. 121 (72.0)	245 (83.9) vs. 169 (80.9)	191 (65.6) vs. 116 (59.2)	99 (35.0) vs. 66 (33.7)	41 (14.0) vs. 17 (8.1)	139 (47.6) vs. 83 (39.7)
So Jin Park et al.	1265 (54.9) vs. 869 (48.4)	879 (38.2) vs. 617 (34.4)	NR	NR	2177 (94.5) vs. 1528 (85.1)	1973 (85.6) vs. 1476 (82.2)	2072 (89.9) vs. 1547 (86.2)	1108 (48.1) vs. 740 (41.2)	NR	895 (38.8) vs. 474 (26.4)
Won-Woo Seo et al.	306 (41.1) vs. 296 (32.4)	73 (9.8) vs. 70 (7.7)	276 (37.1) vs. 125 (13.7)	NR	394 (53.0) vs. 239 (26.1)	701 (94.2) vs. 791 (86.5)	614 (82.5) vs. 726 (79.4)	NR	46 (6.2) vs. 59 (6.5)	168 (22.6) vs. 98 (10.7)
Hiroyoshi Mori et al.	101 (47.9) vs. 101 (47.9)	38 (18.0) vs. 32 (15.2)	136 (64.5) vs. 137 (64.9)	134 (63.5) vs. 122 (57.8)	112 (53.1) vs. 108 (51.2)	197 (93.4) vs. 203 (96.2)	99 (46.9) vs. 92 (43.6)	NR	7 (3.3) vs. 9 (4.3)	45 (21.3) vs. 57 (27.0)

NR: not reported; CCB: calcium-channel blocker; ACEIs/ARBs: angiotensin-converting enzyme inhibitors/angiotensin receptor blockers.

**Table 2:** Clinical outcomes and main characteristics of patients with coronary artery spasms treated with statins or without statins.

Study	Year	Country	Total (*n*)	Statins (*n*)	Age (y^∗^)	Male (*n*, %)^∗^	MACE definition	MACE (*n*)^∗^	Follow-up
Kensuke Io et al.	2007	Japan	1047	129	64 ± 10 (all)	NR	Death from MI, cerebral infarction, or HF and nonfatal MI	3 vs. 31	3.7 y
Sunao Kodama et al.	2009	Japan	292	71	64 ± 10 (all)	NR	Fatal cardiovascular disorder, acute MI, cerebrovascular disorder, HF, aortic aneurysm, renal failure, and obstructive atherosclerosis, undergoing cardiac surgery	3 vs. 13	4.3 y
Munenori Kosugi et al.	2011	Japan	231	87	NR	NR	Sudden cardiac death and readmission for ACS	7 vs. 22	5.9 y
Akihiro Nakagomi et al.	2013	Japan	265	98	59.0 ± 10.4 (all)	NR	Sudden cardiac death and readmission for ACS	5 vs. 26	7.4 y
Masanobu Ishii et al.	2016	Japan	256	128	64.6 ± 9.9 vs. 64.8 ± 9.7	55 (43.0) vs. 57 (44.5)	Cardiac death, hospitalization for acute MI, and unstable angina	0 vs. 9	4.6 y
Min Seok Oh et al.	2016	South Korea	562	281	55.8 ± 9.2 vs. 55.7 ± 9.2	238 (84.7) vs. 241 (85.8)	Cardiac death, MI, any revascularization	9 vs. 12	4.6 y
Zhe Hao Piao et al.	2016	South Korea	501	292	57.4 ± 11.8 vs. 57.9 ± 13.4	201 (69.3) vs. 145 (69.4)	All-cause death, nonfatal MI, and TVR	5 vs. 12	1.0 y
So Jin Park et al.	2019	South Korea	4099	2304	54.9 ± 11.6 vs. 53.4 ± 12.5	1706 (74.1) vs. 1244 (69.3)	Cardiac arrest and acute MI after discharge	120 vs. 97	3.9 y
Won-Woo Seo et al.	2020	South Korea	1658	744	55.9 ± 10.9 vs. 53.5 ± 11.5	461 (62.0) vs. 544 (59.5)	Cardiac death, ACS, and new-onset life-threatening arrhythmia	32 vs. 28	1.9 y
Hiroyoshi Mori et al.	2022	Japan	422	211	65.5 ± 9.5 vs. 64.6 ± 10.3	157 (74.4) vs. 157 (74.4)	Cardiac death, nonfatal MI, hospitalization due to unstable angina pectoris, HF, and appropriate implantable cardioverter defibrillator shock	10 vs. 13	2.6 y

^∗^Statin group vs. no-statin group; age is expressed in average values (SD). MI: myocardial infarction; HF: heart failure; ACS: acute coronary syndrome; TVR: target vessel revascularization.

**Table 3:** Analysis of study methodology and bias of 10 studies included in the meta-analysis of patients with CAS with or without statins using the Newcastle-Ottawa scale.

Study	Representativeness of the exposed cohort	Selection of nonexposed cohort	Ascertainment of exposure	Demonstration that outcome of interest was not present at the start	Comparability of cohorts on the basis of the design or analysis	Assessment of outcome	Suitable length of follow-up	Adequacy of follow-up	Score
Kensuke Io et al.	0	1	1	1	0	1	1	1	6
Sunao Kodama et al.	0	1	1	1	0	1	1	0	5
Munenori Kosugi et al.	0	1	1	1	0	1	1	1	6
Akihiro Nakagomi et al.	0	1	1	1	0	1	1	1	6
Masanobu Ishii et al.	1	1	1	1	1	1	1	1	8
Min Seok Oh et al.	1	1	1	1	1	1	1	1	8
Zhe Hao Piao et al.	1	1	1	1	0	1	0	1	6
So Jin Park et al.	1	1	1	1	0	1	1	1	7
Won-Woo Seo et al.	1	1	1	1	1	1	0	1	7
Hiroyoshi Mori et al.	1	1	1	1	1	1	1	1	8

Each section can score a maximum of 1 except comparability, which can score a maximum of 2. Total score is out of 9.

## Data Availability

The data that support the findings of this study are available from the corresponding author upon reasonable request.
